# ESR Position Paper on Imaging Biobanks

**DOI:** 10.1007/s13244-015-0409-x

**Published:** 2015-05-22

**Authors:** European Society of Radiology (ESR)

**Affiliations:** Neutorgasse 9/2, 1010 Vienna, Austria

**Keywords:** Biomarker, Biobank, Personalised medicine, Quantitative imaging

## Abstract

In March 2014 the European Society of Radiology (ESR) established a dedicated working group (ESR WG on Imaging Biobanks) aimed at monitoring the existing imaging biobanks in Europe, promoting the federation of imaging biobanks and communication of their findings in a white paper. The WG provided the following statements: (1) Imaging biobanks can be defined as “organised databases of medical images and associated imaging biomarkers (radiology and beyond) shared among multiple researchers, and linked to other biorepositories”. (2) The immediate purpose of imaging biobanks should be to allow the generation of imaging biomarkers for use in research studies and to support biological validation of existing and novel imaging biomarkers. (3) A long-term scope of imaging biobanks should be the creation of a network/federation of such repositories integrated with the already-existing biobanking network. The aim of the WG was to investigate the existence, consistency, geographical distribution and type of imaging biobanks in Europe. A survey among ESR members resulted in the identification of 27 imaging biobanks, mostly disease-oriented and designed for research and clinical reference. In 80 % access to imaging biobanks is restricted.

*Key points*

• *Imaging biobanks are “shared databases of imaging biomarkers, linked to biorepositories*”.

• *Exploitation of traditional and imaging biobanks is meaningful for “personalised medicine*”.

• *A European imaging biobank network would significantly boost research in the imaging domain*.

## Introduction

Biobanks are repositories for the storage and retrieval of biological samples of a large number of subjects. A major goal of biobanks is the organised collection of biological material and associated information to spread access among scientists requiring this information [1].

The first biobank project, established in 1948, collecting blood samples and data, was the Framingham Heart Study (FHS), funded by the National Institute of Health-National Heart, Lung, and Blood Institute (NIH-NHLBI) [2]. A recent review performed by Kang et al. reported that 70 % of the world’s biobanks are located in Europe and that the top six countries with biobanks are the UK (*n* = 15), USA (*n* = 14), Sweden (*n* = 12), France (*n* = 9), The Netherlands (*n* = 8) and Italy (*n* = 8) [3].

Until recently, imaging data coming from sources such as magnetic resonance imaging (MRI) or computed tomography (CT) were not included in such biobanks. Over the past 3–4 years, projects have been launched that plan to acquire large repositories of image data, including the UK Biobank and the German National Cohort [4–6].

With the rise of these efforts it is of utmost importance to register and document all existing and new imaging biobanks and provide a structured unified approach for storage of and access to these data from distributed databases. The next challenge will be to reliably connect the available imaging biobanks to tissue biobanks to explore possible imaging biomarkers and provide access to deep phenotypes.

Due to the vast amount of data, imaging biobanks need specific requirements, which have to be considered when setting up the database. Furthermore, imaging data have to be processed to extract *quantitative* information, which might evolve into a so-called “*imaging biomarker*” [7].

Due to the variety of image acquisition methods and sequences, especially in MRI, it is crucial to store detailed image acquisition parameters along with the image data. The large variability in the selection of imaging acquisition parameters in MRI provides a considerable challenge in connecting data from multiple imaging biobanks worldwide. Harmonising data-acquisition protocols as well as standardising image processing methods to extract reliable information will be of great value. The need for harmonisation, validation and standardisation of quantifiable imaging biomarkers in medical imaging has been the motivation to establish the Quantitative Imaging Biomarkers Alliance (QIBA^TM^) initiative of the Radiological Society of North America (RSNA) and the European Imaging Biomarkers Alliance (EIBALL), initiative of the European Society of Radiology (http://www.myesr.org/cms/website.php?id=/en/membership/statutory_committees_working_groups/research_committee/european_imaging_biomarkers_alliance_-_eiball.htm). [8, 9].

In March 2014, the European Society of Radiology also established a dedicated working group (ESR WG on Imaging Biobanks) aimed at monitoring the existing imaging biobanks in Europe, promoting the federation of such imaging biobanks, and elaborated a white paper [10]. Therefore, as part of the working group’s objectives, this article aims to explicate the ESR’s position on the establishment and development of imaging biobanks.

## Imaging biobanks: definition and rationale

Imaging biobanks are defined as organised databases of medical images, and associated imaging biomarkers (radiology and beyond), shared among multiple researchers, linked to other biorepositories [10].

Already existing biobanks are designed to give researchers access to large collections of patient/subject samples and data. Biobanks group biological material of healthy subjects (population-based) and/or patients with specific pathologies (disease-oriented), of which the most frequent are cancer-related. Most biobanks focus only on the collection of genotype data, but do not simultaneously come with a system to collect related clinical or phenotype data. In particular, most biobanks do not include or are not linked to any kind of imaging information, neither primary images nor imaging biomarkers. Comprehensive exploitation of biobanks that also include imaging (genotype and phenotype) is an important cornerstone in diagnostics in the era of “personalised medicine” [10–14].

Personalised medicine describes the intent to provide individual patients with state-of-the-art diagnostic tests, tailored interventions and specific treatments, whenever clinically indicated. Personalised medicine proposes the adjustment/customisation of health care from a one-size-fits-all approach to a patient-specific diagnosis and treatment [15]. As such, personalised medicine can be described as an evidence-supported pre-selection and assignment of tests and therapy selections to patients in need. Quantitative medical imaging, potentially resulting in the discovery of imaging biomarkers, is an essential part of personalised medicine providing a priori selection criteria and a posteriori follow-up strategies, tailored to a given patient with a specific clinical need. Obviously, development of such strategies will be greatly enhanced by the availability of large data repositories [16–19].

Biobanks give researchers access to large repositories of biomaterials for a broad spectrum of further and future analysis, e.g., genetic, genomic, epigenetic, mRNA, proteomics and transcriptomics. The large scale and the broad spectrum of data allow the detection and validation of relevant biomarkers for personalised medicine. Moreover, biobanking in European networks will result in harmonisation of health, lifestyle and other exposure data as well as the development and implemention of harmonised definitions of diseases by increased consensus on the criteria for clinical endpoints.

The classical biobanking activities are to be mirrored by a similar network of imaging biobanks. Modern radiology and nuclear medicine can also provide multiple imaging biomarkers of the same patient, using quantitative data derived from all sources of digital imaging, such as CT, MRI, PET, SPECT, US, x-ray, etc. [20, 21]. Imaging biobanks are infrastructures with massive storage and computing capacity. High-performance computing resources are needed to facilitate image processing comparison, standardisation and validation. Integration of resources and services through a platform that manages the information flow and image processing is a step needed in the development of imaging biobanks.

Other types of images can also be collected from endoscopy, microscopy, surgery, etc., also providing measurable personalised data. All this imaging information should be considered as the phenotypic expression of a patient and can be linked to the genotype. Such data should be available to the research community [22, 23].

A European imaging biobanks network would significantly boost European research in the imaging domain by stimulating the design and validation of new imaging biomarkers, as well as improving our understanding of their biological significance.

This requires standardisation, validation and benchmarking of the data in imaging biobanks. This activity will further stimulate the linking and integration of existing (national and regional) image data repositories as well as the link between imaging biobanks and traditional biobanks.

Standards will have to be developed and implemented. Innovative solutions that promote fair access to high-quality data sets with regard to image-based phenotypes and imaging biomarkers will provide support to users for its utilisation.

Finally, the economic and ethical/legal issues for the management of imaging biobanks have to be explored. These will advance insights and yield benefits to enhance collaborative research, utilise limited resources effectively and share data, technology and expertise. Research on image data management and analysis plays a key role in improving the performance of protocols, software-based analysis and further methodologies for imaging biobanks and the development and validation of imaging biomarkers. All these aspects will surely foster high-level multicenter collaboration.

## Imaging informatics of imaging biobanks

An imaging biobank is a data repository supporting the gathering, querying and dissemination of imaging data for primary or secondary use in research, education and training. It comprises a repository of images and a database to support indexing based on the organ, modality or pathology, etc.

An imaging biobank may be used for different research purposes, for example, defining and validating new imaging biomarkers for diagnosis or prognosis, by comparing them with existing proven (imaging) biomarkers. It may also be used to identify the genetic origin of a disease or factors (including environmental factors) favouring disease development (cohort studies). The images may also be used to build high-quality anatomical templates, tailored to specific populations, e.g., young subjects between 15 and 20 years or patients with Parkinson's disease above 65 years.

General requirements with respect to the data collection are therefore a database facilitating storage of image data and metadata, storage of derived image-based measurements and storage of associated non-imaging data, taking into account the need to deal with longitudinal data and to cope with multiple file formats (DICOM, of course, but also formats used in research and post-processing settings such as NifTI) [24]. With respect to security, functional and technical requirements should be defined with respect to data transformation (including de-identification and encryption), infrastructure (e.g., user identity management, audit log) and data access and movement (including authorisation and transmission protection) [25]. International collaboration will require the advance of both technological and organisational matters (incorporation, procedures, protocols, data sharing, boards and access criteria). This new environment can be considered as a framework on top of a set of computing and data-intensive infrastructures that will provide researchers with tools, protocols, data and expertise to improve medical imaging research and patient health care.

Three main scenarios currently exist in terms of setup:

1. imaging biobanks of clinical research data
2. imaging biobanks with disease-specific data (not necessarily connected to a precise clinical research question)
3. imaging biobanks with general population data

First scenario In this scenario, imaging biobanks are envisaged as infrastructure to archive, share and disseminate (for secondary use) image data that were originally used in the context of clinical research projects, such as clinical trials. In this scenario, the images may have undergone some image processing in order to extract one or more imaging biomarkers relevant to the scientific question that motivated the study. It is natural to store such biomarkers as well as related provenance metadata in the imaging biobank database.
Second scenario In this scenario, imaging biobanks are envisaged as resources to receive, archive, share and disseminate images in specific clinical domains, e.g., Alzheimer’s disease or multiple sclerosis. Such systems aim at collecting the clinical images of patients with a given pathology on a broad scale (e.g., national scale). They usually address common specific image acquisition protocols and ensure quality control at the time of image importation to ensure optimal image use. These imaging biobanks can also be based on a regional or national screening initiative collecting image data of a group of persons with specific characteristics such as habits, previous disease or a genetic profile. Breast cancer, lung cancer and colon cancer screenings are examples of image-based screening with subsequent storage of images in an imaging biobank.
Third scenario In this scenario, imaging biobanks are composed of data collections obtained from the general population without a specific research goal or disease-oriented approach. Longitudinal data are collected over a long period of time with as much data collected of as many subjects as possible. Research projects may gain paid access to this data upon request for a specific research question. The technical setup of this scenario is similar to the second scenario, but the data collection is not performed for clinical purposes and will in many cases be acquired by entities established especially for the data collection of the population study.

## Services provided by imaging biobanks

1. **Management of image collections**: This involves several aspects: (a) negotiating the conditions of image acceptance and conservation: duration of conservation, formats, image quality, sharing with owners, sharing with other parties, general and specific (i.e., collection-dependent) metadata, ability to link to external data (clinical information, biological information, biological specimen); (b) defining metadata associated with a new image collection; (c) applying workflow when a new image is added to a collection (receiving images from a source and assessing image quality, recording information about data acquisition and acquisition devices, assessing proper data selection according to the specific research project, recording additional information besides the images such as measurements, functional data).
2. **Supporting image collections**’ **consultation**: This includes sending study requests to a review committee that determines the scientific merit and checks for similar requests that were previously granted. If approved, the committee sets up access rights at the desired level to the selected subset of the data and supports query/retrieval processes, i.e., processing queries from external parties, verifying access rights, querying the database, providing references of images and related metadata, and downloading images.
3. **Relating image cases to external data** (**data**, **specimen**): This involves: (a) receiving notifications from external parties, (b) cross-referencing identifiable information to connect the two databases and determine subjects for which this is feasible and reliable enough, and (c) updating the database with the references of external data.
4. **Production of imaging biomarkers**: This service requires the prior definition of a process to validate the imaging biomarker to guarantee to the requesting party that the measurements performed are accurate. Standardisation and registration of the process should also be part of this. The biomarkers’ calculation service includes: (a) applying the relevant image-processing workflow, (b) recording the calculated values in the database as well as (c) the detailed provenance data (by whom, how and when such data were obtained).

## Federated access to imaging biobanks

A key feature is to provide end-users with large federated systems (e.g., through a web portal or a set of web services), facilitating data query from multiple imaging biobanks. This avoids users querying all the imaging biobanks successively that they believe might contain the kind of data they are looking for. Providing such service involves:

1. setting up a catalogue of image collections provided by multiple imaging biobanks;
2. striving towards a “federated data model” for databases querying (e.g., using common data schemas, standard nomenclatures, reference to common ontologies);
3. receiving queries from external parties;
4. querying the distributed databases according to the “federated data model”;
5. providing image metadata matching the query, including references to image data. These federated systems will improve health care through studies of quality control, such as image quality and radiation dose, technical and protocol comparisons, follow-up assessment of clinical guidelines, fast translation of research findings into image-guided management, assessment of image biomarker prognostic factors and early assessment of treatment response.

## Clinical focus

Imaging biobanks should be focussed on collecting data from healthy subjects or—disease-oriented—from patients (oncologic imaging, rare diseases, other). In the case of healthy subjects (as in the UK Biobank), it should be considered how these data can be collected to ensure participant safety and identity protection. In principle, the imaging acquisition should minimise or avoid the use of ionising radiation with greater dependence on US and MRI in healthy subjects. In contrast, disease-related IB can also be collected by x-ray and (ultra) low-dose CT, represented by data derived from screening programmes for breast cancer (x-ray mammography) and colon and lung cancer (CT colonography and low-dose lung CT) [26–30]. Furthermore, developments in CT imaging with ultra-low-dose acquisitions could open the possibility of CT imaging in population screening for subjects above a certain age.

Oncologic imaging seems to be the easier setting for the collection of imaging biomarkers from multiple imaging modalities. Oncology patients routinely undergo multiple imaging studies for staging and follow-up of the disease to evaluate the TNM and the treatment response. The same patients undergo multiple laboratory tests, pathologic specimen analysis (which provides further imaging data), genetic sequencing, etc. In this setting it seems highly relevant to link imaging biomarkers derived from clinical investigation with those collected by traditional biobanking [31].

Clinical research will benefit from the increased production of scientific researchers' contributions from the field of radiology.

## Economics, standards, security, legal issues

### Economic

The creation and realisation of biobanks is a very expensive process because of the involvement of a large number of participants, e.g., the UK Biobank with about 500,000 participants or the nationwide biobank in Iceland. Imaging procedures as part of prospective cohort studies will contribute to the overall costs in a relevant portion, especially if MR imaging is necessary because of the research focus and radiation exposure, contrast media or medication with other modalities (e.g., cardiac CT).

In large studies, dedicated imaging centres have been established or are in planning. Based on the example of the UK, it has been shown that the costs per participant would be approximately €500; however this would end up at about €50 M for the imaging part based on the assumption of 100,000 participants [32].

### Standards

Standards are relevant for different levels, e.g., legal regulation, organisational issues for the institution, standard operating procedures for handling of data and samples, but also for the syntax and semantic (ontologies) representation in information systems.

In the context of medical imaging, there are three major topics:

- *Reporting*: there is a common trend to structured reporting worldwide [33]. Automated evaluation of information in databanks would work best with standardised documentation of findings and measurements. There are relatively mature solutions providing the platform for such efforts. The Integrating the Healthcare Enterprise (IHE) profile, “Managing Radiology Report Templates”, describes the use of appropriate templates. Recommendations for workflows and implementations are available, while first implementations are under evaluation [33].
- *Semantic Interoperability*: Imaging procedures for imaging biobanks will very often rely on quantitative imaging biomarker data, to be exchanged between systems. Suitable models should therefore be defined to describe them in an explicit and consistent way, ideally using semantic models such as ontologies (e.g., SNOMED, RadLex, ICD).
- *Imaging and communication formats*: due to the worldwide acceptance of DICOM there are minor issues in the topic of imaging formats. DICOM has ongoing revised and new requirements, e.g., new imaging techniques or ontologies will be included into the standard. Workflows and communication across institutions are almost covered by IHE profiles. Based on the IHE Cross-Enterprise-Communication concept (IHE-XDS family), which is the core of many regional and national eHealth concepts, there is already a logical approach for connecting imaging centres with imaging biobanks. These IHE-XDS concepts would allow decentral storage of primary data, but also the aggregation of data, and also communication across different imaging biobank domains (www.ihe.net). Actually, there is a missing link between the imaging world and the information about biobanks. The description of requirements and workflows should be followed by the development of an appropriate IHE profile for these use cases [34, 35].

## Security and legal issues

Security and legal issues are two of the most challenging tasks in building large research databases in general. This is due to the requirements for the probable long-term aggregation of medical data from different sources linked with personal information on one side and the request for patient privacy and security aspects on the other. Such databases might contain personal data and information, and also digitised collections of specimens such as tissues or blood, genomic and imaging data.

Many of such research-oriented databases are built as legacy systems with proprietary regulations, regulated by different levels of privacy rules, for example [36, 37].

There are some examples of concepts for the legal frameworks for biobanking; one of these is from the German “Telematics Platform of Medical Research Network” (TMF) [38]. One of the relevant requirements is a solution for interdisciplinary and active collaboration between different institutions, for which the property rights and privacy of the “donors” (information data and/or biological material from volunteers and patients) have to be protected, even for scientific and also possible commercial use by third parties.

Therefore different legal requirements have to be considered [39]:

- *Legal status of the institution* organising and maintaining the databank.
- *Property rights* of samples and information, will often require explicit transfer of rights by the donor.
- *Relevance of medico*-*legal and professional regulations*, e.g., the acquisition of samples is usually a medical act, which requires interaction with a physician, even in a research setting. Another topic is a regulation about the communication on new findings while in research or data analysis, which might impact the health of the donor.
- *Continuity* of databanks in case of ending projects or insolvence of the institution;
- *Secure transfer* of samples or information and *secure storage*;
- *Confiscation protection and research secrecy*.

The complexity in building large databanks for international collaboration may increase in case of different national regulations. The actual development at the European level with the preparation of a General Data Protection Regulation (COM (2012) 0011) will also be relevant for the future of imaging biobanks [40].

There is an interest in unified worldwide legislation regarding privacy issues and ownership, also to require mandatory registration of biobanks.

## Imaging biobanks in Europe: results of the ESR survey

One of the aims of the working group was to investigate the existence, consistency, geographical distribution and type of imaging biobanks in Europe.

A survey was carried out, using a free online poll service (www.surveymonkey.com), inviting heads of radiology departments across Europe to answer the following nine questions: purpose of the biobank, types of cases, number of cases, kind of imaging data, presence of follow-up image examinations, already existing publications on the image biobank, accessibility of the image biobank (open, restricted to user), imaging data supported and image format.

The results of the questionnaire are summarised in Table 1.

**Table 1:** Results of the ESR survey on imaging biobanks

Purpose of the biobank	Number (percentage)
Research	14 (82.4)
Clinical reference	12 (70.6)
e-learning	9 (52.9)
Other	1 (5.9)
Types of cases available	
Oncologic	13 (76.5)
Cardiovascular	8 (47.1)
Healthy volunteers	7 (41.2)
Rare diseases	6 (35.3)
Other	6 (35.3)
Number of cases available	
Less than 300	5 (29.4)
300–500	3 (17.6)
500–1000	3 (17.6)
1000–2000	1 (5.9)
More than 2000	5 (29.4)
Kind of imaging data available	
Computed tomography	14 (82.4)
Magnetic resonance	15 (88.2)
Hybrid imaging	5 (29.4)
Ultrasound	9 (52.9)
Other	5 (29.4)
Availability of follow-up image examinations	
Not available	5 (29.4)
Available	12 (70.6)
Availability of publications based in the image biobank	
Not available	12 (70.6)
Available	5 (29.4)
Accessibility to the biobank	
Fully open on the Internet	1 (6.3)
Open to any registered user (free registration)	2 (12.5)
Restricted to users involved in predefined projects	5 (31.3)
Restricted to the personnel of the local department/hospital	8 (50.0)
Imaging data supported	
Strictly limited to acquired images	5 (31.3)
Also contains processed images (segmentation, registration, etc.)	8 (50.0)
Also contains imaging biomarkers	3 (18.8)
Image formats	
Strictly limited to DICOM	13 (81.3)
DICOM and other common formats used in research (such as NIFTI, Analyze)	1 (6.3)
Also contains imaging biomarkers	2 (12.5)

Twenty-seven responses were received from Austria, Finland, France, Germany, Kazakhstan, Italy, Russia, Slovenia, Spain, Sweden, Switzerland, The Netherlands, Turkey and the UK.

Most biobanks are for research and clinical reference and are disease-oriented (oncologic and cardiovascular type of cases); the number of cases included is extremely variable, but mostly less than 1000, and most biobanks contain CT and MR images.

Of note, the accessibility of the biobanks is in the vast majority (80 %) restricted to registered users involved in predefined projects or personnel of the local department/hospital.

## Conclusion

Medical imaging biobanks are virtual biobanks recently emerging for advancing the study of rare, cardiovascular, oncologic and neurological diseases, the identification of early biomarkers and surrogates, and the development of population studies. These biobanks will evaluate the impact of new quantitative biomarkers in early disease diagnosis, disease phenotyping, disease grading, targeting therapies and evaluation of disease response to treatment.

Such imaging biobanks are currently at an early stage of development and dissemination within Europe. The survey carried out among heads of radiology departments across Europe proved the existence and operation of some disease-oriented patient- or healthy population-based image databases. Most of them were developed within institutional projects and are not freely accessible. The working group elaborated the rationale, definition, technical, clinical, economics, management, legal and ethical issues related to the setup, development, federation and maintenance of imaging biobanks. As the imaging biobanks are focussing on the phenotype expression, there is an unmet need to link the information to other biomarkers from other sources of information, especially all biobanks. Therefore the working group endorsed the integration of imaging biobanks with already traditional biobanks (genomic, tissue and other type of “omics”), since the joint exploitation of both biobanks is an important cornerstone for diagnosis and targeted therapies in the era of personalised medicine.
